# Fragments of the Bacterial Toxin Microcin B17 as Gyrase Poisons

**DOI:** 10.1371/journal.pone.0061459

**Published:** 2013-04-10

**Authors:** Frédéric Collin, Robert E. Thompson, Katrina A. Jolliffe, Richard J. Payne, Anthony Maxwell

**Affiliations:** 1 Department of Biological Chemistry, John Innes Centre, Norwich Research Park, Norwich, United Kingdom; 2 School of Chemistry, The University of Sydney, New South Wales, Australia; Institut National de la Recherche Agronomique, France

## Abstract

Fluoroquinolones are very important drugs in the clinical antibacterial arsenal; their success is principally due to their mode of action: the stabilisation of a gyrase-DNA intermediate (the cleavage complex), which triggers a chain of events leading to cell death. Microcin B17 (MccB17) is a modified peptide bacterial toxin that acts by a similar mode of action, but is unfortunately unsuitable as a therapeutic drug. However, its structure and mechanism could inspire the design of new antibacterial compounds that are needed to circumvent the rise in bacterial resistance to current antibiotics. Here we describe the investigation of the structural features responsible for MccB17 activity and the identification of fragments of the toxin that retain the ability to stabilise the cleavage complex.

## Introduction

DNA gyrase and DNA topoisomerase (topo) IV are enzymes belonging to the DNA topoisomerase family, responsible for the regulation of DNA topology in all cells [1], [2]. They have been very successfully exploited as targets for fluoroquinolones and other antibacterials since they are not present in mammalian cells [3]. However, with the rise of bacterial resistance to fluoroquinolones and other current antibiotics, new inhibitors are needed. DNA gyrase has the unique function of introducing negative supercoils into closed-circular DNA [4], whereas topo IV is responsible for decatenation and relaxation of closed-circular DNA [5]; both gyrase and topo IV require ATP hydrolysis for activity. The success of the fluoroquinolones is due to the fact that they stabilise a transient topoisomerase-DNA covalent complex that can lead to the lethal release of broken DNA [6]. This intermediate is referred to as the cleavage complex: a covalent adduct where both strands of the DNA have been cut and linked to the enzyme, thus forming a gate through which another segment of DNA can be passed (the strand-passage event) to alter DNA topology. Inhibitors stabilising the cleavage complex are often referred to as topoisomerase poisons. Other antibacterials that inhibit gyrase by different mechanisms are referred to as catalytic inhibitors. These include the aminocoumarins, such as novobiocin, that compete with ATP binding [7] and simocyclinone D8, a related antibiotic that prevents DNA from binding to the enzyme [8]. There are a limited number of bacterial toxins that are known to stabilise the gyrase-DNA cleavage complex: among them are the protein CcdB [9], [10] and the peptide toxin microcin B17 [11].

Microcin B17 (MW 3093 Da) was initially isolated from strains of *Escherichia coli* [12]; the expression of the toxin was later linked to the plasmid pMccB17 that carries the *mcb* operon [13], [14]. This operon is comprised of seven genes: *mcbA*, the structural gene, encodes a peptide that is post-translationally modified by the microcin synthases (*mcbB*, *mcbC*, *mcbD*), which convert serine and cysteine residues into oxazole and thiazole rings within the peptide sequence [15]. The remaining genes, *mcbE*, *mcbF*, *mcbG*, encode proteins responsible for export and immunity. Previous work has shown that these heterocycles are critical, and modifications of these residues proved to be detrimental to the antibacterial activity of MccB17 [16]. When MccB17 was first identified, it was reported to be active on a variety of organisms (*Escherichia*, *Enterobacter*, *Pseudomonas* and *Shigella*) [12], however its activity *in vitro* has only been characterised with *E. coli* gyrase [11], [17]. In spite of its attractive antibacterial properties, the poor physico-chemical properties of MccB17 prevent it from being a good drug candidate. The mode of action of MccB17 is unknown, but its ability to stabilise the cleavage complex is strongly enhanced by the presence of ATP [11] suggesting that toxin activity is linked with the strand-passage event.

In order to understand the mode of action of MccB17, it is necessary to determine the features of the molecule that are required to promote the stabilisation of the gyrase-DNA cleavage complex. Therefore, fragments of MccB17, ranging from the heterocyclic amino acids present in the toxin to peptides covering specific regions of the MccB17 sequence, were generated. This was achieved through a combination of degradation reactions on MccB17 and its analogues and chemical synthesis. The evaluation of these species demonstrated that fragments under 2 kDa are capable of retaining the cleavage-complex stabilisation activity of MccB17. These MccB17 fragments may serve as tools to uncover the mechanism of the natural product toxin as well as potential starting points for the development of new gyrase inhibitors.

### Notations

Fragments of MccB17 described in this work are denoted as intervals of residues: Mcc[first residue-last residue]. The residues are numbered according to preMccB17, the translational product of *mcbA*. As an example, full-length MccB17 is denoted as Mcc[Val27-Ile69]. The following abbreviations are used for the heterocyclic residues: H-**oz**-OH: 2-aminomethyloxazole-4-carboxylic acid; H-**oztz**-OH: 2-[2′-aminomethyloxazole-4′-yl]thiazole-4-carboxylic acid; H-**tz**-OH: 2-aminomethylthiazole carboxylic acid; H-**tzoz**-OH: 2-[2′-aminomethylthiazole-4′-yl]oxazole-4-carboxylic acid. Heterocyclic residues are referred to according to the first residue of their precursor sequence. As an example the first heterocyclic residue in MccB17 would be denoted oztz39 from its precursor Gly39-Ser40-Cys41.

## Results

### Activity of small fragments of MccB17

**Figure 1:**
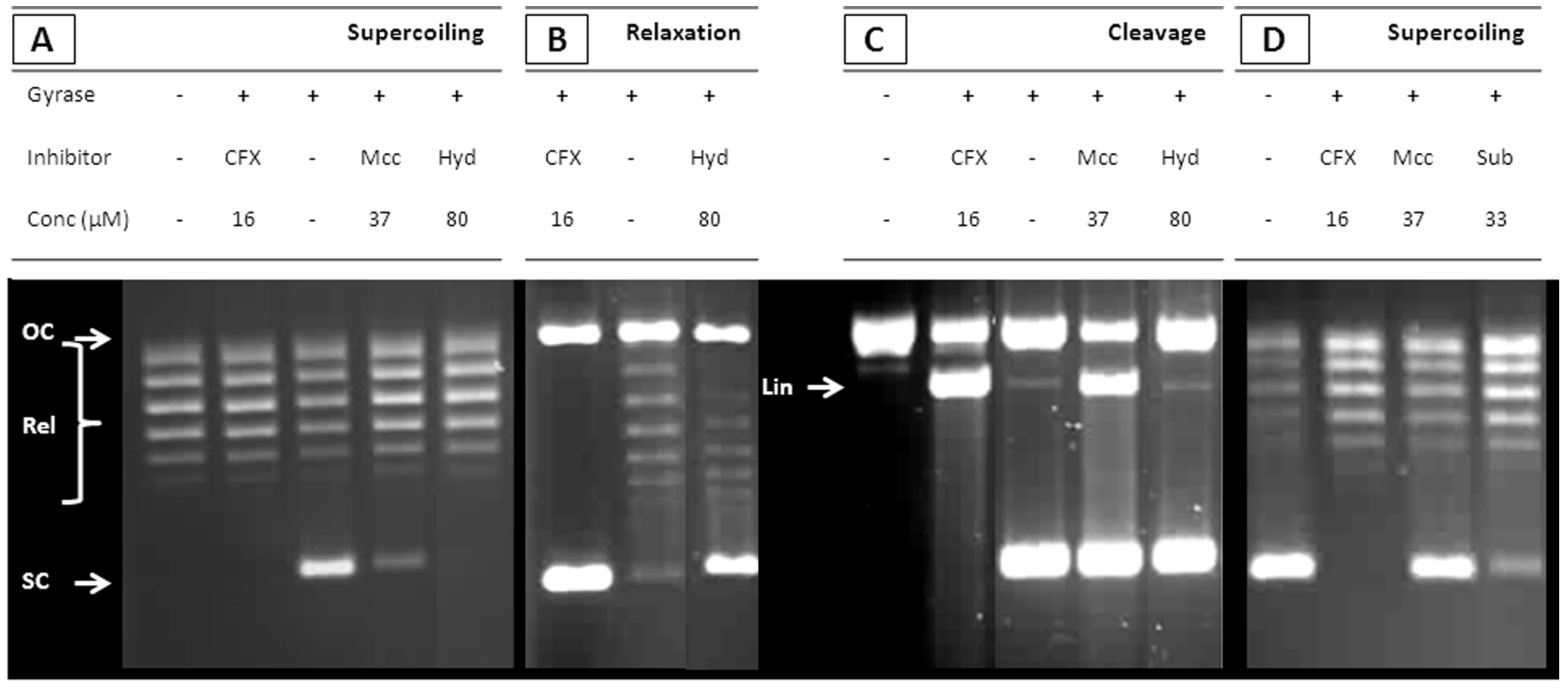
Evaluation of the activity of digested MccB17 mixtures. Effect of the alkaline hydrolysate (Hyd) of MccB17 on supercoiling (A), relaxation (B) and cleavage (C) assays with *E. coli* gyrase. The DNA species are annotated as follow: OC (open circular), Rel (relaxed), SC (supercoiled), and Lin (linear). Activity is compared with ciprofloxacin (CFX) and MccB17 (Mcc). (D) Effects of a mixture resulting from the digestion of MccB17 by subtilisin (Sub) in a supercoiling assay with *E. coli* gyrase compared to controls without inhibitors, and with ciprofloxacin (CFX) and MccB17 (Mcc). All reactions contain 3.3% DMSO.

In order to generate low molecular weight fragments of MccB17, recombinantly produced MccB17 was hydrolysed with aqueous sodium hydroxide solution; the mixture obtained after neutralization and lyophilisation was tested for inhibitory activity on DNA gyrase. Supercoiling (Figure 1A) and relaxation (Figure 1B) reactions are completely abrogated at 80 µM of hydrolysed MccB17, but no stabilisation of cleavage complex is observed (Figure 1C). Despite extensive efforts, the identity of the component(s) responsible for the inhibitory activity of the mixture could not be established.

**Figure 2:**
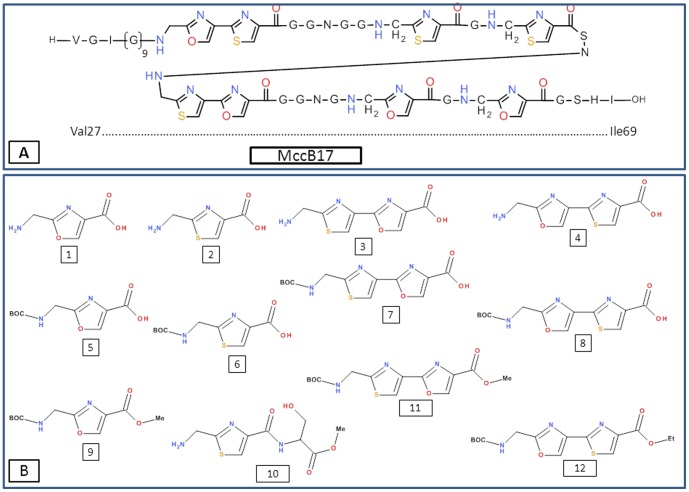
Structure of MccB17 and synthetic heterocyclic compounds. (A) Structure of MccB17, the first and last residues are numbered. (B) The structures of the synthetic compounds related to MccB17 are shown: monoheterocyclic compounds (1; 2; 5; 6; 9; 10) displayed low to no inhibitory activity on gyrase supercoiling at 1 mM; bis-heterocyclic structures (3; 4; 7; 8; 11; 12) show inhibition between 50 and 500 µM with 3 being the most active. None of the compounds stabilise the gyrase-DNA cleavage complex.

Analysis by LC-MS of the alkaline hydrolysate obtained above showed that it contained significant amounts of the heterocyclic amino acids present in MccB17. Therefore, the activity of these modified amino acids on gyrase was evaluated. Four heterocyclic derivatives (Figure 2B: 1–4) were synthesized as described [18]. The heterocyclic amino acids, together with synthetic intermediates (Figure 2B) were tested both for their ability to inhibit *E. coli* gyrase supercoiling and to stabilise the gyrase-DNA cleavage complex. However, gyrase supercoiling is inhibited by the various compounds only at high concentrations (0.5–1 mM) and no stabilisation of the cleavage complex occurred, indicating that small heterocyclic amino acids are not effective inhibitors of gyrase activity and that these components are not responsible for the inhibitory activity in the hydrolysate noted above. We therefore turned our attention to the investigation of larger fragments of MccB17.

### Proteolysis of MccB17 by subtilisin

**Figure 3:**
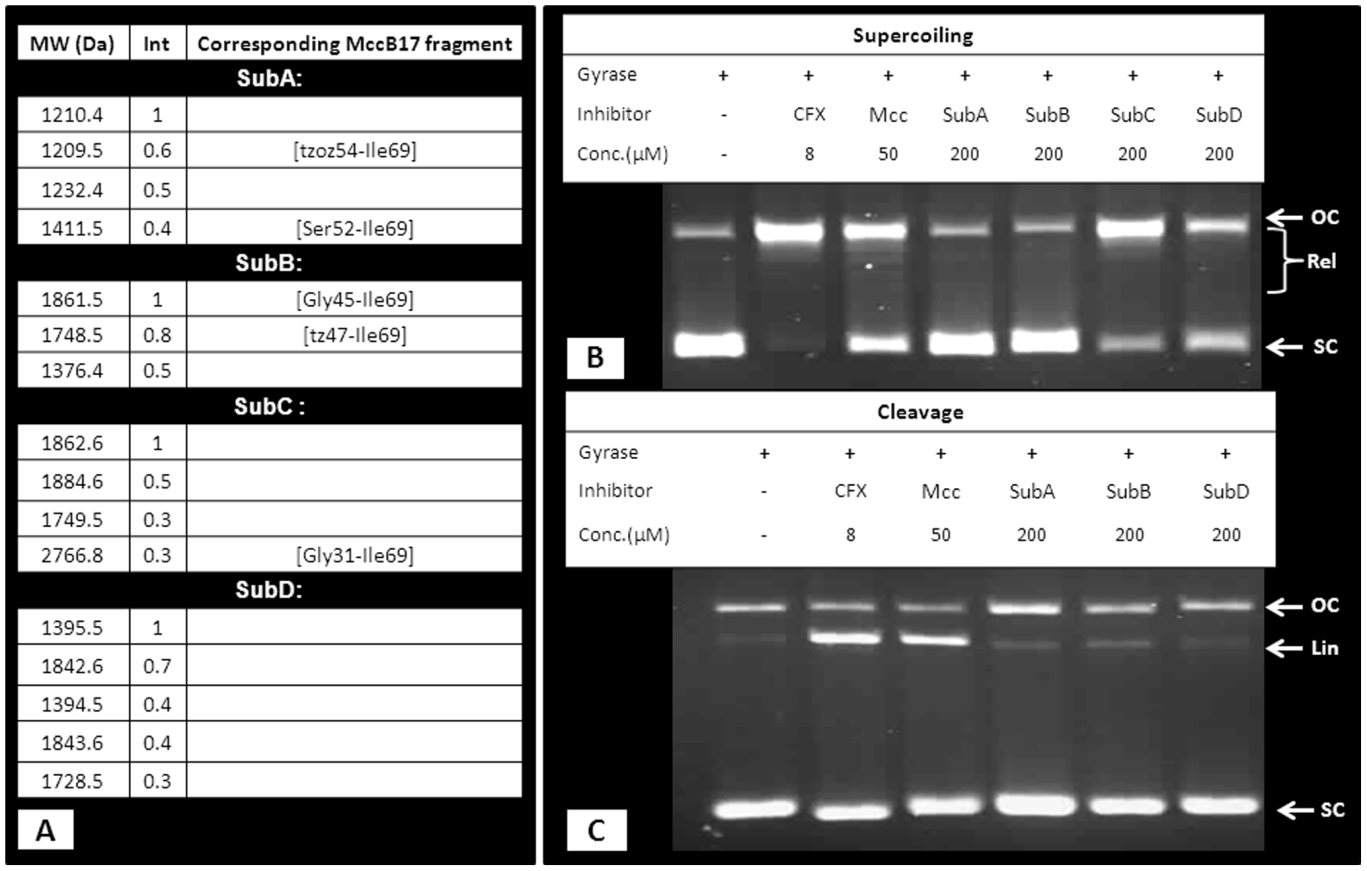
Evaluation of HPLC fractions from MccB17 proteolysis by subtilisin. (A) M+H ions observed for each HPLC fractions (cf Figure S2) with relative intensity (Int). HPLC fractions SubA to SubD were tested in supercoiling assays (B). Fractions SubA, SubB, SubD were tested on cleavage-complex stabilisation assays (C); fraction SubC was not assayed due to the presence in the mixture of Mcc[Gly31-Ile69], which was known to stabilise the cleavage complex. The DNA species are annotated as follow: OC (open circular), Rel (relaxed), SC (supercoiled), and Lin (linear).

In early work by Asensio and Perez-Diaz, the sensitivity of MccB17 to proteolysis by subtilisin was reported [12], and the resulting mixture exhibited a loss of antibacterial activity. There is a possibility that this loss of activity was due to the inability of the proteolysis products to reach their target inside the organism, having lost their affinity for MccB17 transporters (OmpF and SbmA). To address this question we next evaluated the *in vitro* activity of MccB17 digested by subtilisin. MccB17 was incubated overnight with subtilisin at 37°C and the mixture was purified by reverse-phase chromatography and analysed by MALDI-ToF MS. In the mixture, MccB17 had been completely digested, and fragments resulting from the specific cleavage were the main products. However, compounds resulting from non-specific degradation are present as well (Figure S1). The resulting proteolytic mixture inhibits gyrase in supercoiling assays (Figure 1D). In order to identify the active component(s), the mixture was fractionated by HPLC. The four fractions eluting after 45 min were collected for evaluation (Figure S2) as our preliminary (unpublished) work had shown that active components eluted after that retention time. Analysis of these fractions by MALDI-ToF MS shows that they all contain several fragments of MccB17, some with side-chain deamination (Figure 3A). Fractions SubC and SubD show significant inhibition of supercoiling at a concentration of 200 µM (Figure 3B) and display some stabilisation of the cleavage complex, albeit weak. Surprisingly, fraction SubB, which did not have a strong inhibitory activity on supercoiling, shows weak stabilisation of the cleavage complex (Figure 3C).

### Proteolysis of MccB17 by trypsin

**Figure 4:**
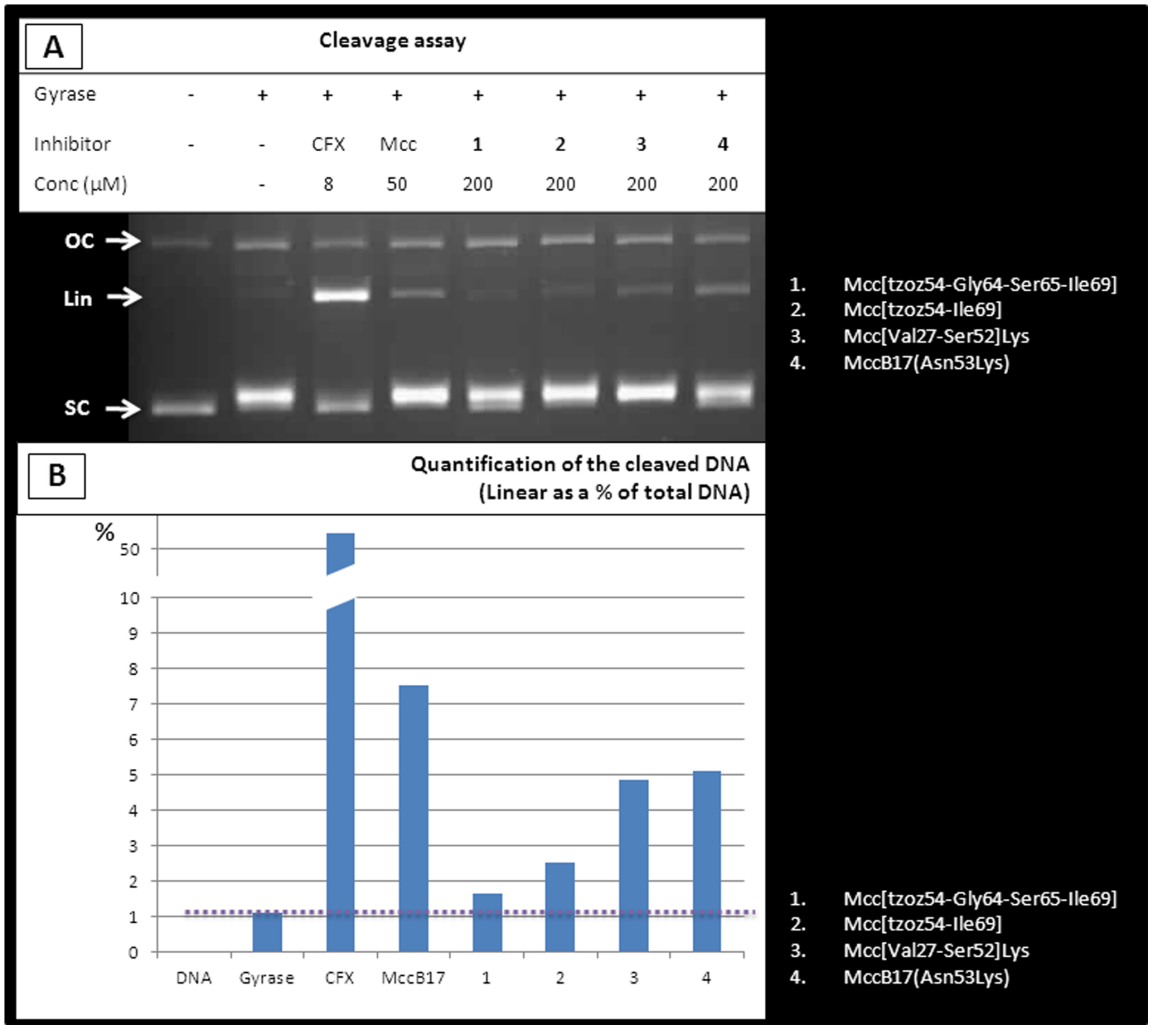
Evaluation of peptides derived from MccB17. (A) Cleavage-complex stabilisation assay of peptides resulting from MccB17(Asn53Lys) digested with trypsin (Mcc[Val27-Ser52]Lys and Mcc[tzoz54-Ile69); ciprofloxacin (CFX), MccB17, and MccB17(Asn53Lys) were included for comparison, as well as a side-product corresponding to Mcc[tzoz54-Ile69] lacking one oxazole ring: Mcc[tzoz54-Gly64-Ser65-Ile69]. The DNA species are annotated as follow: OC (open circular), SC (supercoiled), and Lin (linear). The cleaved DNA band for each compound was quantified, the results are summarized in (B), which presents the amount of cleaved DNA as a percentage of the entire DNA present in the corresponding lane. The mutated MccB17 and the two resulting fragments stabilise the cleavage complex: MccB17(Asn53Lys) and Mcc[Val27-Ser52]Lys show about 2/3rds of MccB17 activity, whereas Mcc[tzoz54-Ile69] has about 1/3rd. Mcc[tzoz54-Gly64-Ser65-Ile69], which is Mcc[tzoz54-Ile69] lacking an oxazole ring as a cleavage-complex stabilisation activity close to baseline (shown as a dotted line).

MccB17 does not naturally contain any lysine or arginine residues and, as such, would not normally be a substrate for trypsin digestion. Therefore we engineered MccB17 to introduce a lysine residue at a specific site. Digestion of a lysine-containing MccB17 generates two fragments: the N-terminal fragment in which the last residue is substituted by a lysine, and the corresponding C-terminal fragment of native MccB17. We created the mutation Asn53→Lys, as the digested products would lead to the smallest fragment of MccB17 observed in the subtilisin digest fraction able to stabilise the cleavage complex (fraction SubA, Figure 3). As anticipated, the digestion of the modified MccB17 with trypsin led to the expected two fragments as main products Mcc[Val27-Ser52]Lys and Mcc[tzoz54-Ile69], which were subsequently separated by HPLC. Both fragments were tested in gyrase cleavage assays and surprisingly both of the fragments are able to weakly stabilise the cleavage complex (Figure 4).

### MccB17 derivatives lacking terminal residues

**Figure 5:**
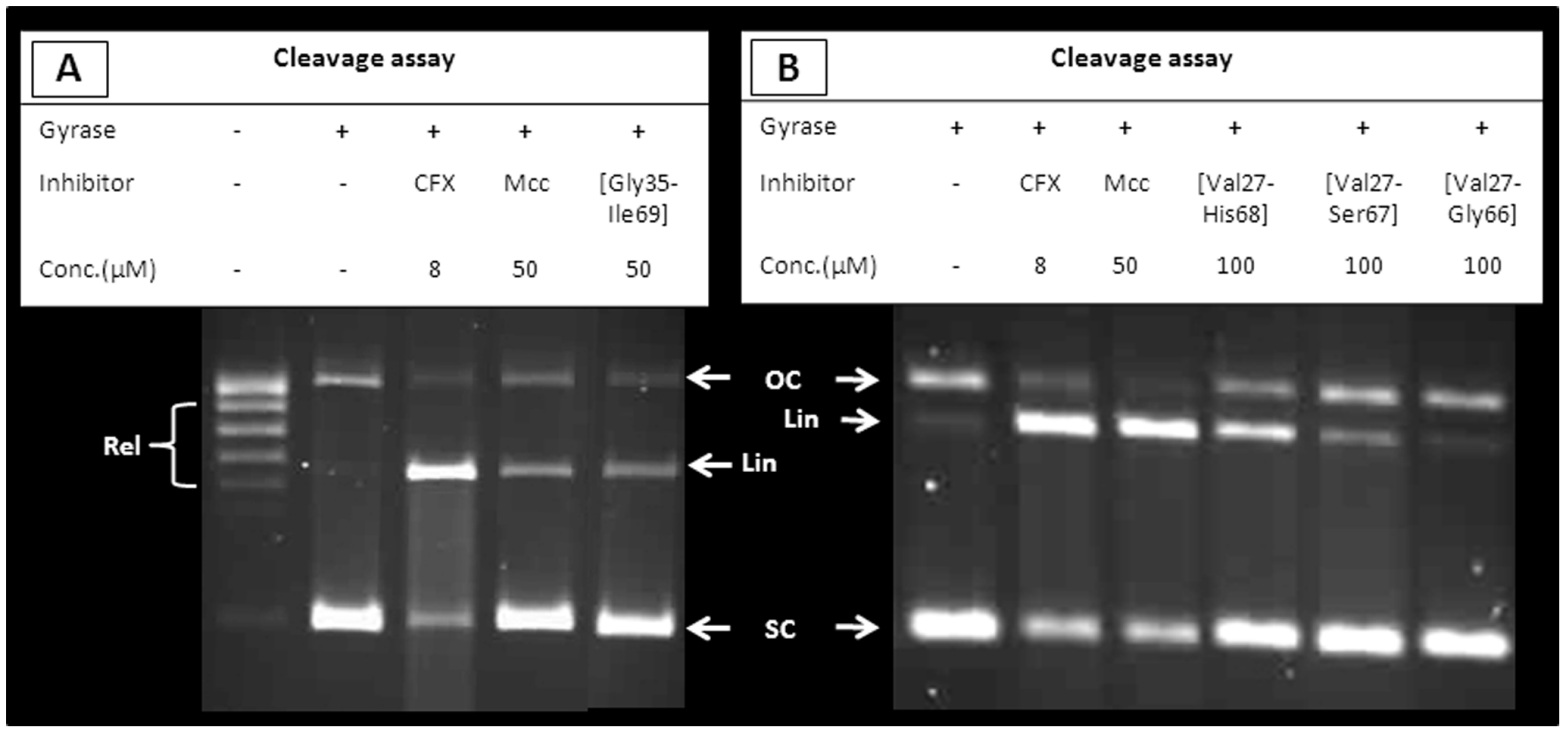
Removal of the N- or C-terminal amino acids of MccB17. Comparison of the cleavage-complex stabilisation activity on *E. coli* gyrase between native MccB17 (Mcc) and MccB17 lacking the first 8 amino acids Mcc[Gly35-Ile69], (A); or MccB17 lacking 1, 2 or 3 C-terminal amino acids (respectively Mcc[Val27-His68]; Mcc[Val27-Ser67]; Mcc[Val27-Gly66]), (B). The DNA species are annotated as follow: OC (open circular), Rel (relaxed), SC (supercoiled), and Lin (linear). The activity is not altered by the removal of N-terminal residues (Mcc[Gly35-Ile69]) whereas removal of C-terminal residues causes the loss of the ability to stabilise the cleavage complex, with Mcc[Val27-Gly66] having no activity.

The determinants for MccB17 activity were further investigated by assessing whether the residues from the extremities of the toxin are essential. MccB17 derivatives lacking N-terminal amino acids were generated following the same strategy used with the MccB17 tryptic digest: substitution of Gly30, Gly32 or Gly34 by a lysine, expression of the modified toxin and removal of the N-terminal domain by tryptic digest. In a similar fashion, species lacking C-terminal residues were generated by replacing the codon of a chosen C-terminal residue by a stop codon, thus eliminating all the residues downstream. The first strategy was applied successfully to engineer MccB17 species lacking up to 8 N-terminal residues (Mcc[Gly35-Ile69]) by mutating Gly34→Lys in the polyglycine tail and digesting the expressed peptide with trypsin. MccB17 species lacking Ile69, His68-Ile69, or Ser67-His68-Ile69 from the C-terminus were isolated after introducing stop codons. All truncated MccB17 analogues were evaluated in supercoiling and cleavage assays. Only the cleavage assay results are shown here as they are more significant for the antibacterial activity, but similar trends were observed with supercoiling. Mcc[Gly35-Ile69], the derivative lacking eight N-terminal residues is able to stabilise the cleavage complex at the same level as native MccB17 (Figure 5A), whereas MccB17 species lacking C-terminal residues show a strong decrease in their ability to stabilise the cleavage complex as residues are removed from the C-terminus; with Mcc[Val27-Gly66] losing all cleavage-complex stabilisation activity (Figure 5B).

### Synthetic fragments of MccB17

**Figure 6:**
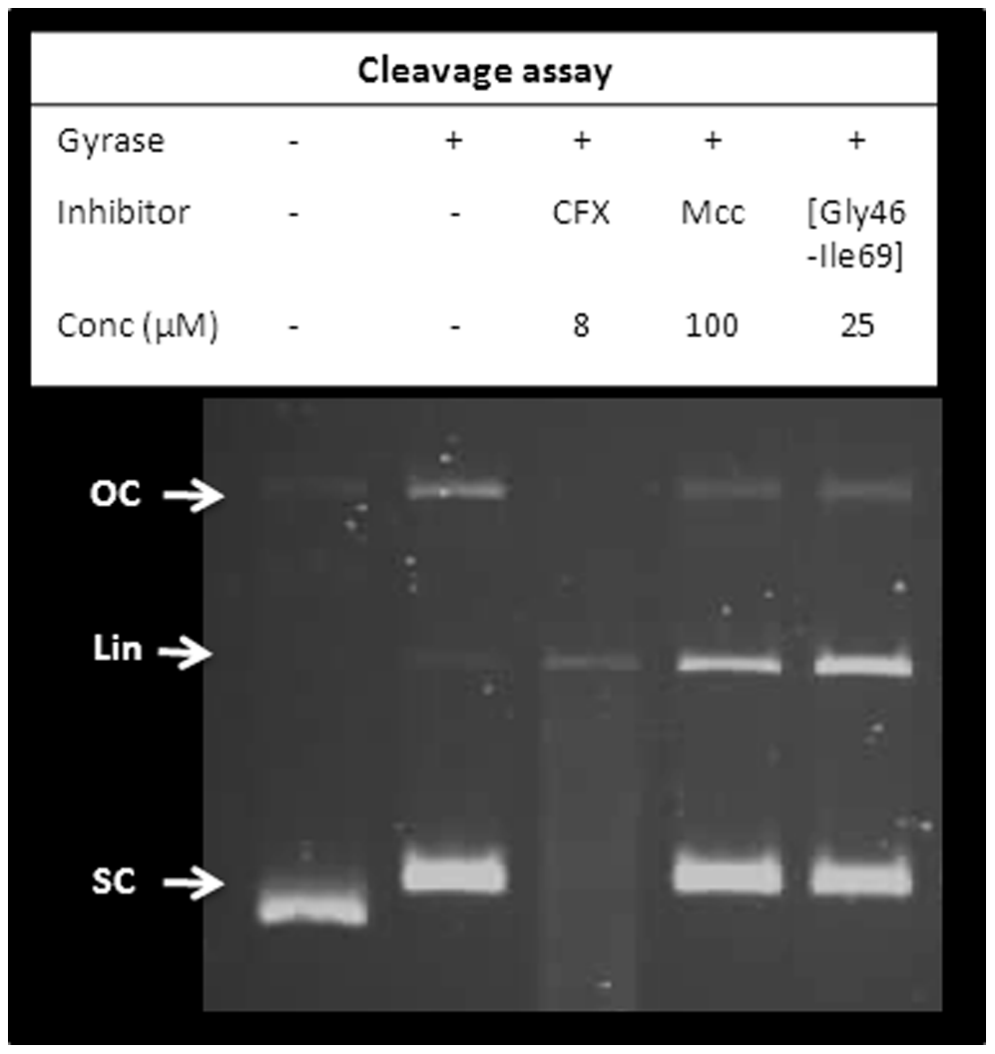
*E. coli* gyrase cleavage assays with synthetic fragments of MccB17. At 25 µM Mcc[Gly46-Ile69] shows cleavage-complex stabilisation activity similar to MccB17 at 100 µM, whereas Mcc[oz61-Ile69] did not show any activity (not shown). The DNA species are annotated as follow: OC (open circular), SC (supercoiled), and Lin (linear).

A convenient ligation-based method for the total synthesis of MccB17 has recently been reported [19], which involves the assembly of three synthetic fragments to form MccB17. The availability of these fragments has enabled the evaluation of MccB17 fragments of various lengths for their gyrase activity. To this end, Mcc[oxz61-Ile69], Mcc[Gly46-Ile69] as well as the full-length synthetic MccB17 were tested in supercoiling and cleavage assays. The short C-terminal fragment Mcc[oxz61-Ile69] has no inhibitory activity (data not shown) whereas Mcc[Gly46-Ile69], a longer fragment, has strong inhibitory activity on gyrase supercoiling and strongly stabilises the cleavage complex (Figure 6). As expected, synthetic MccB17 has activity comparable to natural MccB17.

The synthetic compounds were also tested in halo assays against two strains of *E. coli* (Table 1): MG1655 a standard strain, and NR698 a permeable strain (due to a compromised outer membrane that makes it more susceptible to various chemical agents [20]). Synthetic MccB17 shows inhibition of growth similar to the natural toxin, whereas the synthetic fragments Mcc[Gly46-Ile69] and Mcc[oz61-Ile69] do not inhibit the growth of bacteria in either strain, suggesting that smaller fragments are not able to cross the inner membrane.

**Table 1:** Halo assays of synthetic fragments of MccB17.

Compound	Halo diameter (mm)		DNA cleavage
	MG1655	NR698	at 100 µM
**CFX (120 µM)**	17.6	25.4	1.0
**Bio MccB17 (1.5 mM)**	14.6	15.7	0.3
**Chem MccB17 (1.5 mM)**	13.2	12.7	0.4
**Mcc[Gly46-Ile69] (1.5 mM)**	0	0	0.5
**Mcc[oz61-Ile69] (1.5 mM)**	0	0	0.0
**DMSO**	0	0	0.0

The table summarises the diameter of the halos observed with the deposition of 2 µl of drug in DMSO on a lawn of 10^9^ MG1655 or NR698 cells after overnight incubation. The quantification of the amount of linear DNA observed for each compound in cleavage-complex stabilisation assays is shown normalized to the amount of linear DNA obtained with ciprofloxacin. Bio MccB17 = toxin purified from bacteria; Chem MccB17 = synthesised toxin.

## Discussion

Microcin B17, an antibacterial natural product, has a chemical structure shaped by several factors: its ability to stabilise the gyrase-DNA cleaved intermediate (which is directly linked to its bactericidal properties), the requirement of its biosynthesis and in particular its post-translational modifications, the necessity of the producing bacteria to protect itself against its own toxic agent, and finally the uptake of the toxin by the targeted organism. In this work we have highlighted features linked directly to the gyrase poisoning activity and, by extension, features associated with the maturation of the toxin. We have shown that a large section of the N-terminus of MccB17 can be removed without altering its properties, whereas alteration of the C-terminus leads to loss of the cleavage-stabilising activity. This is consistent with work by Sinha Roy *et al* [21], who demonstrated that the polyglycine present in the N-terminus was a spacer necessary for the processing of the heterocyclic moieties. Here we demonstrate that this area of the toxin is not required for the gyrase-poisoning activity thereby confirming that most of the N-terminus of MccB17 is present only to anchor the microcin synthase and enable the formation of the thiazole and oxazole moieties. Going further, we demonstrate that fragments resulting from the proteolysis of MccB17, down to half its size retain a weak ability to stabilise the gyrase-DNA cleavage complex. These results encouraged us to evaluate synthetic fragments of MccB17; molecules down to MW = 1.8 kDa (Mcc[Gly46-Ile69]) proved to be as potent *in vitro* as the native toxin. However halo assays carried out with this synthetic fragment suggest that smaller fragments might not be recognised by the uptake system in the bacteria, but it is feasible that such small fragments may be taken up by other mechanisms.

**Figure 7:**
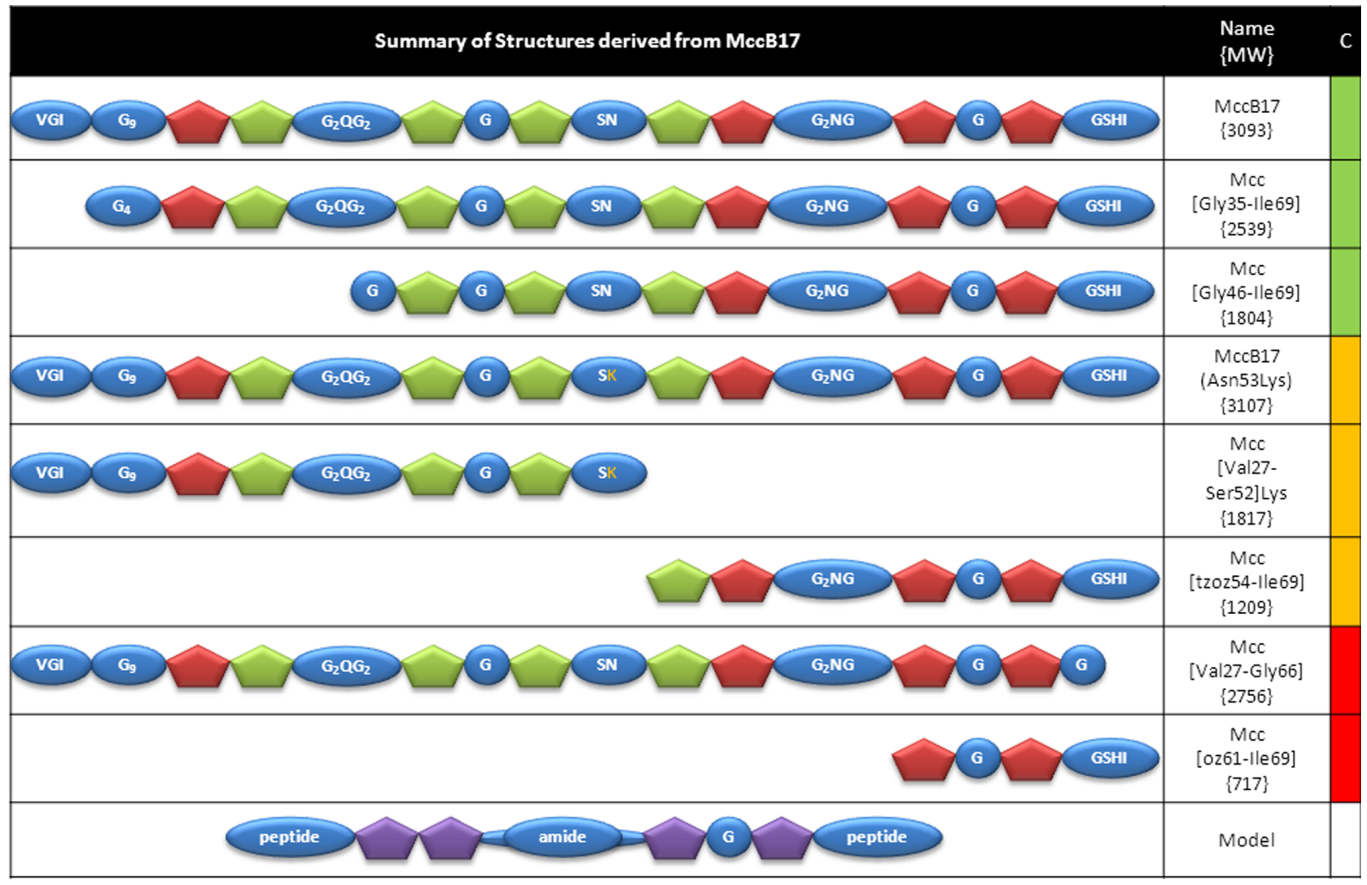
Comparison of MccB17 derivatives and fragments. Summary of the fragments of MccB17 studied in this work. Sequences, names used in this study, and qualitative stabilisation of cleavage are displayed. The cleavage activity (CA) is colour-coded: green, same level as MccB17; orange, low level of cleavage observed; red, no cleavage observed. The bottom schematic structure is the proposed model template for new gyrase poisons.

Among the MccB17 derivatives that have been studied in this work, Mcc[Gly46-Ile69] is of particular interest from a primary structure point of view, as it lacks the polyglycine sequence and a bis-heterocycle without alteration of the cleavage activity. Apart from the Gly residues, MccB17 is comprised of an alternation of heterocyclic moieties, polar residues (Asn and one Ser) and a Ser-His-Ile tail. Furthermore, the arrangement of these polar and aromatic residues could be viewed as a repeat of two motifs with the following sequence: peptide, bisheterocycle, amide-containing spacer, monoheterocycle-Gly-monoheterocycle, peptide (Figure 7). Following this idea it could be assumed that each unit may have some inherent activity, but the linkage positions them for optimal potency. This hypothesis is supported by the low cleavage-complex stabilisation activity observed with the separate halves of MccB17 obtained with trypsin cleavage, and furthermore with the strong activity of Mcc[Gly46-Ile69], which contains this motif preceded by two additional thiazoles and a Ser-Asn linker. On the other hand, the fact that we observe a loss of activity by altering the C-terminal Ser-His-Ile of MccB17 would suggest that these residues contribute to important interactions or that the shift of position of the terminal carboxylate caused by their removal is detrimental. Two questions arise from these observations: first, from a practical point of view, could this motif be a model (Figure 7) to develop new gyrase poisons? The size of this template makes it suitable for a combinatorial approach, i.e. combinations of different terminal peptides and heterocyclic content could be explored in order to optimize the interaction with the target, the physico-chemical properties, and to affect gyrase from other pathogens. Second, is the question of the role of this motif in the toxin's mode of action: what is the relevance of the dimeric nature of MccB17 in the context of the complex with the gyrase-DNA intermediate? The stoichiometry of MccB17 binding to gyrase is unknown, and its action has directly been linked with strand passage [17], [22]: the conformational changes occurring during this catalytic event may create the MccB17 binding site. Considering this motif repeat in the context of the open C-gate might be of significance for future modelling studies.

### Conclusions

In this work we have provided information about the structural features of MccB17 that are linked to gyrase poisoning, however the details of the molecular interaction remain unknown. Structural studies would provide such details and the active MccB17 derivatives we have described here would be suitable substitutes for the native toxin in such approaches: lacking the polyglycine they would be simpler structures to analyse by NMR and potentially better ordered for analysis by crystallography, which will be the subject of future work.

## Materials and Methods

### Strains, plasmids, and reagents

*E. coli* DH5α containing the pUC19-mcb plasmid was provided by Christopher T. Walsh (Harvard Medical School, Boston, USA). *E. coli* GyrA and GyrB were produced as described [23] or purchased from Inspiralis Ltd (Norwich, UK). Relaxed and supercoiled pBR322 plasmids were purchased from Inspiralis Ltd. Subtilisin (Protease from *Bacillus licheniformis*, Type VIII) was purchased from Sigma (P5380) and trypsin (Sequencing Grade Modified Trypsin) was purchased from Promega (V5111). PCR primers were purchased from Sigma-Genosys and PfuTurbo DNA Polymerase was purchased from Agilent Technologies.

### Production of MccB17

Microcin B17 and related mutants were produced from DH5α(pUC19-mcb) following the method described [16]. The raw MccB17 solution was purified on a Dionex Ultimate 3000 HPLC system with a preparative reverse phase column ACE 5 C18-300 250×21.2 mm (ACE-221-2520). Crude MccB17 in DMSO (1 mM) was loaded onto the HPLC via a 5 ml loop and eluted with a linear gradient from 13% to 23% CH_3_CN in water containing 0.1% TFA, over 40 min. The acetonitrile was removed under vacuum prior to lyophilisation.

### Alkaline hydrolysis of MccB17

2.5 ml (2.3 µmol) of a 0.9 mM solution of MccB17 in DMSO, 2.5 ml MQ water, and 0.16 ml of aqueous 5 M NaOH (345 µmol), were introduced into a 10 ml round bottom flask equipped with a stirrer and a condenser. The mixture was stirred at 120°C for 7 days, water was added to compensate for evaporation. The solution was cooled, and the pH adjusted to 7. The mixture was diluted in water to reach 1/40 DMSO in water, and freeze dried.

### Gyrase assays

Supercoiling reactions were carried out as described previously [24]: 30 µl reaction mixtures contained 45 mM Tris.HCl (pH 7.5), 44 mM KCl, 4 mM MgCl_2_, 4 mM DTT, 0.2 mM EDTA, 1.8 mM spermidine, 0.2 mM ATP, 8.5% v/w glycerol, 0.1 g/l BSA), 0.5 µg relaxed pBR322, and 4 nM gyrase, 3.3 % DMSO. The inhibitors were added as 1 µl of a solution in DMSO. The reaction mixtures were incubated for 2 h at 25°C.

DNA cleavage reactions were carried out as for supercoiling with the following changes: 10× more gyrase was used, the reaction mixture was incubated for 30 min at 37°C, then SDS (0.2% w/v) and proteinase K (2 µg/µl) were added and the reaction was incubated for an extra 30 min at 37°C.

Relaxation reactions were carried out under similar condition as supercoiling but omitting ATP and spermidine in the reaction mixture and in the presence of 3× more gyrase.

### Proteolysis reactions

Digestion of MccB17 by subtilisin was conducted as the follows: 0.5 mM MccB17, 10% DMSO, 10 mM NaAcO, 5 mM Ca(AcO)_2_), 0.075 mg/ml subtilisin was incubated at 37°C for 48 h before being loaded on a strata C18_EC QSPE reverse phase column equilibrated with 30 ml acetonitrile and 30 ml MQ water. The column was eluted with 30 ml MQ water and 30 ml 50% acetonitrile/MQ water. This last fraction was collected and further purified by HPLC with a linear gradient of 5 to 23% CH_3_CN in water containing 0.1% TFA, over 80 min. Four fractions were collected.

Lysine-containing MccB17 was digested with trypsin following the supplier's directions: 6 µM of modified MccB17, 20 µg/ml trypsin, 1% DMSO in supplied trypsin buffer for 2 h at 37°C. Trypsin was deactivated by 5 min incubation at 100°C.

### Site-directed mutagenesis

The *E. coli* strain used in this study had been designed by Milne *et al.* [25]: *E. coli* DH5α carrying a pUC19 plasmid containing the MccB17 operon *mcbABCDEFG* (pUC19-*mccB17*). This plasmid was extracted and used as a template for site-directed mutagenesis studies. The MccB17-coding sequence was amplified by PCR using forward (F) and reverse (R) primers carrying the desired mutation:

Mcc[Gly34Lys](F): tggtggtggcaaaggcggcggcg; Mcc[Gly34Lys](R): cgccgccgcctttgccaccacca; Mcc[Ile69STOP](F): tcacattaatgatacgttgaattaa; Mcc[Ile69STOP](R): ttaattcaacgtatcattaatgtgaa; Mcc[His68STOP](F): aagtggttcataaatctgata; Mcc[His68STOP](R): tatcagatttatgaaccactt; Mcc[Ser67STOP](F): ggaagtggttaacatatctgata; Mcc[Ser67STOP](R): tatcagatatgttaaccacttccg; MccB17[Asn53Lys](F): tggtggtgcagcaaaggttgtagt; MccB17[Asn53Lys](R): actacaacctttgctgcaaccacca.

The following conditions were used: Pfu Turbo buffer (1×), 1 mM dNTP, 20 nM of F-primer, 20 nM R-primer, 1 µl 0.05 U Pfu turbo, 1 µg/µl pUC19-*mccB17*. 25 µl of the amplification mixture were added to 5 µl of NEB-buffer n°4, 2 µl of DpnI (40 units), and 18 µl of MQ water and incubated overnight at 37°C in order to remove the pmcb template. 7 µl of the resulting mixture was used to transform DH5α high efficiency *E. coli* cells. The transformed cells were grown on LB-ampicillin (0.1 mg/ml) agar overnight at 37°C. The mutagenesis was confirmed by picking colonies, miniprep of the plasmid and sequencing using M13 primers.

### Synthesis of MccB17 analogues

MccB17, Mcc[Gly46-Ile69], and Mcc[oz61-Ile69] were synthesized via a combination of Fmoc-strategy solid-phase peptide synthesis and ligation chemistry as reported previously [19].

## Supporting Information

Figure S1 **MALDI-ToF spectrum of MccB17 digested by subtilisin.** The mixture resulting from the digest of MccB17 by subtilisin was subjected to MALDI-ToF MS analysis. Peaks corresponding to the expected fragments are highlighted; the spectra show that MccB17 has been completely digested (no peak at M+H^+^ = 3094 Da).
(PPTX)

Figure S2 **Fractionation of MccB17 subtilisin proteolysis mixture by HPLC.** The trace corresponding to the elution of the subtilisin digest with a gradient of acetonitrile 0.24% CH_3_CN/min in H_2_O, 0.1%TFA is shown. The fractions collected for evaluation (A–D) are highlighted in blue.
(PPTX)
