# Supplementary figures and images for: Fragments of the Bacterial Toxin Microcin B17 as Gyrase Poisons
Source: PLoS One. 2013 Apr 10;8(4):e61459. doi: 10.1371/journal.pone.0061459 (PMC3622597; doi:10.1371/journal.pone.0061459)

## Slide 1
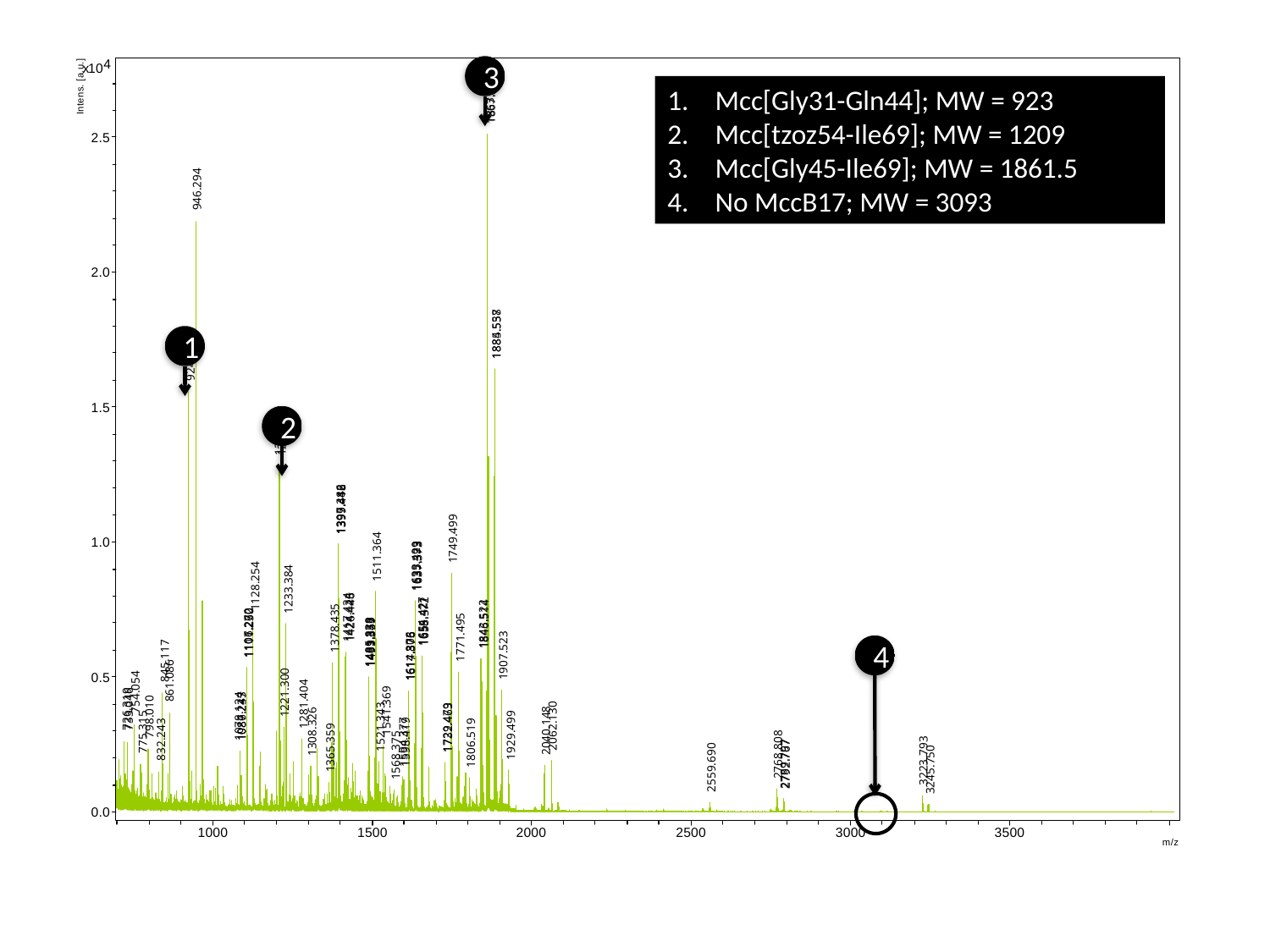

3
Mcc[Gly31-Gln44]; MW = 923
Mcc[tzoz54-Ile69]; MW = 1209
Mcc[Gly45-Ile69]; MW = 1861.5
No MccB17; MW = 3093
1
2
4

Supplement: Figure S1 — MALDI-ToF spectrum of MccB17 digested by subtilisin. The mixture resulting from the digest of MccB17 by subtilisin was subjected to MALDI-ToF MS analysis. Peaks corresponding to the expected fragments are highlighted; the spectra show that MccB17 has been completely digested (no peak at M+H+ = 3094 Da). (PPTX) [file pone.0061459.s001.pptx]

## Slide 1
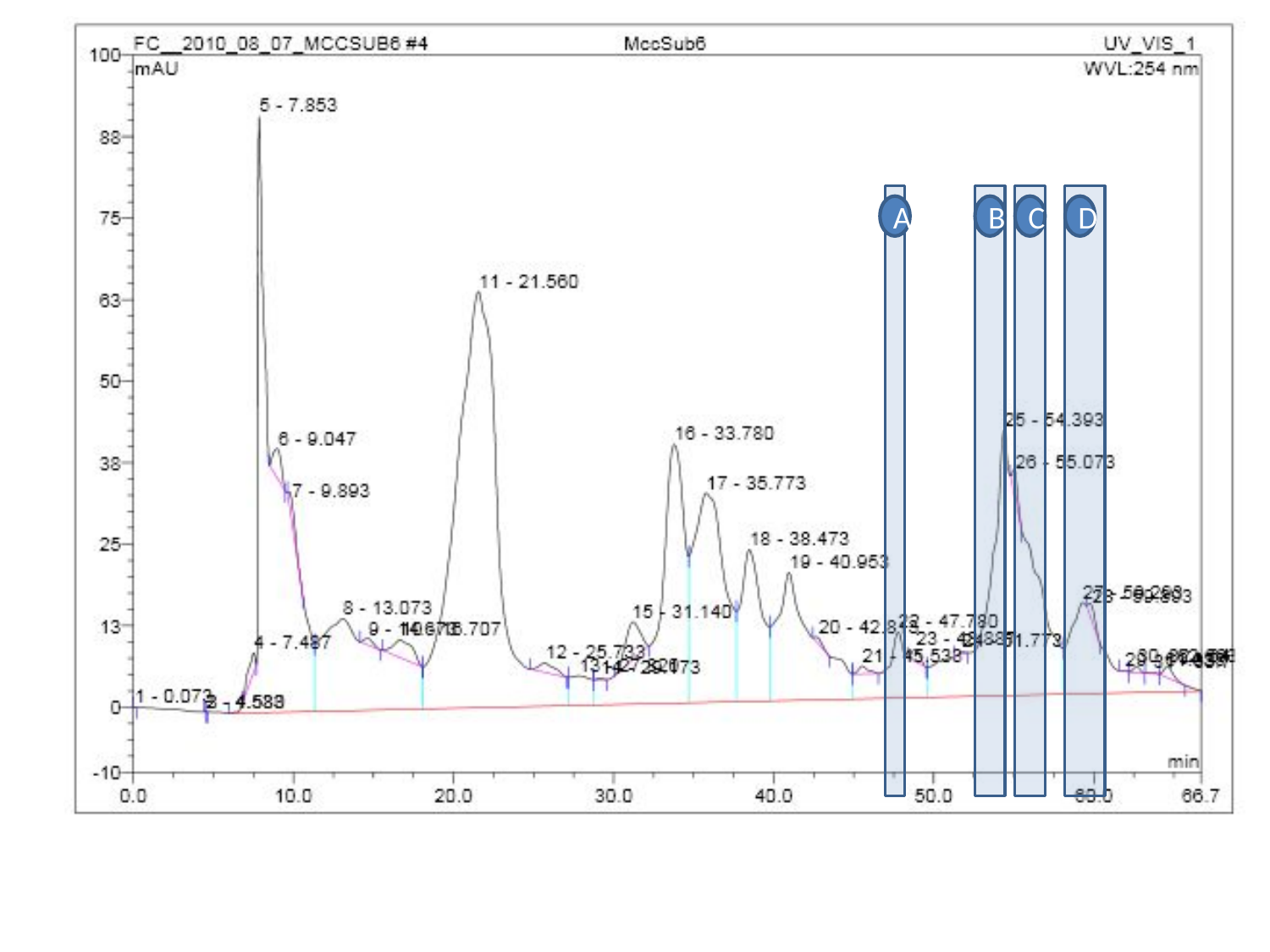

A
B
C
D

Supplement: Figure S2 — Fractionation of MccB17 subtilisin proteolysis mixture by HPLC. The trace corresponding to the elution of the subtilisin digest with a gradient of acetonitrile 0.24% CH3CN/min in H2O, 0.1%TFA is shown. The fractions collected for evaluation (A–D) are highlighted in blue. (PPTX) [file pone.0061459.s002.pptx]
